# 2D-echocardiography vs cardiac MRI strain: a prospective cohort study in patients with HER2-positive breast cancer undergoing trastuzumab

**DOI:** 10.1186/s12947-021-00266-x

**Published:** 2021-11-09

**Authors:** Nathalie I. Bouwer, Crista Liesting, Marcel J. M. Kofflard, Jasper J. Brugts, Marc C. J. Kock, Jos J. E. M. Kitzen, Mark-David Levin, Eric Boersma

**Affiliations:** 1 Department of Internal Medicine , Albert Schweitzer Hospital, 3300 AK Dordrecht, The Netherlands; 2 Department of Cardiology, Albert Schweitzer Hospital, Dordrecht, The Netherlands; 3 Department of Cardiology, Erasmus MC, University Medical Centre Rotterdam, Rotterdam, The Netherlands; 4 Department of Radiology, Albert Schweitzer Hospital, Dordrecht, The Netherlands

**Keywords:** HER2-positive breast cancer, Trastuzumab, Cardiac MRI, Speckle tracking echocardiography, Strain imaging

## Abstract

**Background:**

We aimed to study the predictive value of early two-dimensional echocardiography (2DE) speckle tracking (ST) for left ventricular ejection fraction (LVEF) changes during trastuzumab treatment for HER2-positive breast cancer.

**Methods:**

HER2-positive breast cancer patients receiving trastuzumab, with or without anthracycline, underwent 2DE-ST at baseline and after 3 and 6 months (m) trastuzumab. Cardiac magnetic resonance (CMR) imaging (with ST) was performed at baseline and 6 m. We studied the correlation between 2DE-ST- and CMR-derived global longitudinal strain (GLS) and global radial strain (GRS) measured at the same time. Additionally, we associated baseline and 3 m 2DE-ST measurements with later CMR-LVEF, and with cardiotoxicity, defined as CMR-LVEF < 45% and/or absolute decline > 10% during trastuzumab.

**Results:**

Forty-seven patients were included. Median baseline LVEF was 60.4%. GLS measurements based on 2DE-ST and CMR showed weak correlation (Pearson’s *r* = 0.33; *p* = 0.041); GRS measurements were uncorrelated (*r* = 0.09; *p* = 0.979). 2DE-LVEF at baseline and 3 m, and 2DE-ST-GLS at 3 m were predictive of CMR-LVEF at 6 m. In contrast, the change in 2DE-ST-GLS at 3 m was predictive of the change in CMR-LVEF at 6 m, whereas the change in 2DE-LVEF was not. Importantly, the 11 patients who developed cardiotoxicity (28%) had larger 2DE-ST-GLS change at 3 m than those who did not (median 5.2%-points versus 1.7%-points; odds ratio for 1% difference change 1.81, 95% confidence interval 1.11–2.93; *p* = 0.016; explained variance 0.34).

**Conclusions:**

Correlations between 2DE-ST and CMR-derived measurements are weak. Nevertheless, ST-measurements appeared useful to improve the performance of 2DE in predicting LVEF changes after 6 m of trastuzumab treatment.

**Supplementary Information:**

The online version contains supplementary material available at 10.1186/s12947-021-00266-x.

## Background

Patients with HER2-positive breast cancer receiving trastuzumab treatment are prone for developing cardiac dysfunction, which usually represents as a left ventricular ejection fraction (LVEF) decline. Early identification of cardiac dysfunction is important, as further LVEF reductions or development of congestive heart failure may be prevented by cardio-protective treatment with beta-blockers and/or angiotensin converting enzyme (ACE) inhibitors, or by timely interruption of trastuzumab [1, 2]. However, accurate and widely available cardiac monitoring methods are still in development.

Cardiac magnetic resonance (CMR) imaging is the gold standard for evaluation of the cardiac function. CMR has a low inter-reader variability compared to two-dimensional (2DE) or three-dimensional echocardiography (3DE) with respect to LV function and volumes, which is important for serial follow-up [3]. However, the use of CMR for cardiac monitoring of breast cancer patients is hampered by its limited availability and because CMR is experienced by patients as a burdensome procedure. 2DE might be a reasonable, widely available and more readily accepted alternative in this context. Unfortunately, several studies in a variety of patients showed only poor-to-moderate correlation between 2DE and CMR in measuring the LVEF [4, 5]. This could potentially be improved by adding speckle tracking (ST) to 2DE. With 2DE-ST, strain imaging can be performed which is a sensitive imaging modality that provides opportunities for detecting subclinical cardiac dysfunction in patients receiving cancer therapy [6, 7]. Although global longitudinal stran (GLS) has a moderate intervendor variability, its reproducibility is superior to LVEF measurements and therefore it can be suitable for longitudinal cardiac monitoring [8, 9].

Studies that investigated 2DE-ST and CMR showed moderate to good correlations ranging from 0.50 to 0.89 for GLS, 0.58 to 0.60 for global radial strain (GRS) and 0.51 to 0.92 for global circumferential strain (GCS) in healthy subjects and in patients with a variety of cardiovascular diseases [10–17]. However, most studies did not differentiate between specific cardiovascular diseases. Subsequently, correlations were not consistent among all subgroups [17]. More importantly, patients who were treated with potential cardio-toxic anti-cancer treatment were not included in these studies. Therefore, validation of these correlations is necessary in these specific populations. Furthermore, a growing number of studies have investigated the clinical relevance of strain measurements in patients during anti-cancer treatment. These studies showed that a GLS decline is related to a LVEF decline measured both with the same methods [18–21]. However, the association between early 2DE-ST strain and later (gold standard) CMR-based LVEF has not been investigated extensively, which is important in determining the additional value of strain imaging along with LVEF evaluation in patients during trastuzumab treatment.

Therefore, the goal of the current study was to investigate the correlation and agreement between 2DE-ST strain and CMR strain, and the association between early 2DE-ST strain measurements and subsequent CMR-derived LVEF in patients with HER2-positive breast cancer during trastuzumab treatment.

## Methods

### Study design and participants characteristics

This prospective, observational cohort study included women with HER2-positive early-stage and advanced-stage breast cancer, who underwent trastuzumab treatment from June 2012 until June 2016 in a large teaching hospital in the Netherlands. Patients were excluded from the study in case of baseline CMR-LVEF < 45%, ischemic heart disease, valvular heart disease, severe renal dysfunction, hepatic dysfunction or other contraindications for receiving trastuzumab treatment.

In patients with early-stage breast cancer, trastuzumab was preceded by 4 courses of anthracycline. In patients with advanced-stage breast cancer, trastuzumab was administrated once every 3 weeks until relapse of breast cancer or until the development of cardiotoxicity (for definition see below) [22].

The study was approved by the institutional review board of the hospital (WOAC Albert Schweitzer Hospital), and conducted according with the Declaration of Helsinki. All participants provided written informed consent for their participation in the study, and for the study-related measurements.

### Echocardiography protocol

**Fig. 1:**
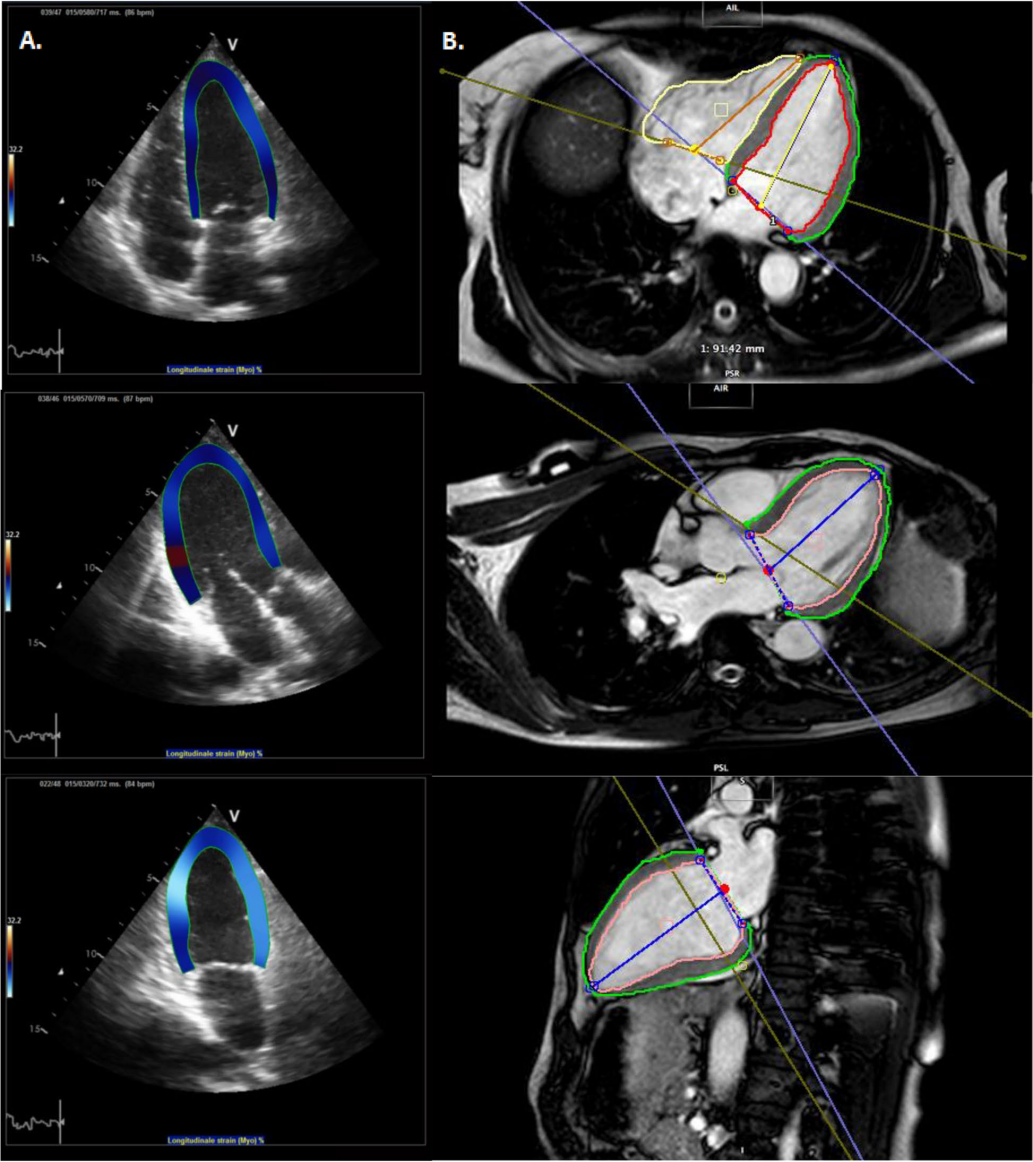
2D-STE and CMR images used for calculation of myocardial strain. Abbreviations: *CMR* cardiac magnetic resonance imaging, *2D-STE* two-dimensional speckle tracking echocardiography. **A**. Speckle tracking analysis with 2D-STE of apical 4-chamber, 3-chamber and 2-chamber view. **B**. Speckle tracking analysis with CMR of transaxial 4-chamber, 3-chamber and sagittal 2-chamber view

2DE was performed at the following time points: before the start of anthracycline (in early-stage patients only), before the start of trastuzumab, after 3-months (m) trastuzumab and after 6 m trastuzumab (Fig. 1, Supplementary). 2DE acquisition was performed on a Vivid 7 echocardiography system (GE Vingmed Ultrasound, Trondheim, Norway). End diastolic volume (EDV) and end systolic volume (ESV) were calculated using Simpson’s biplane method. The LVEF was determined as the difference between EDV and ESV, relative to the EDV. Baseline measurement were for early-stage patients before the start of anthracycline and for advanced-stage patients before the start of trastuzumab. Strain imaging analyses were then performed using validated tracking algorithm software (TomTec Cardiac Performance Analysis version 4.3 CPA, Unterschliessheim, Germany). EDV and ESV were automatically calculated using traced endocardial borders. These borders that were also used to calculate the GLS and GRS were manually drawn and checked by two experienced observers (Fig. 1). GLS was calculated by averaging the values of peak systolic strain of all 6 segments of the 4-, 3- and 2-chamber views. The shortening of the myocardium related to its original length is described by the negative strain values of GLS. GRS was calculated by averaging the peak systolic strain values in all 6 segments of the parasternal short-axis view at midpapillary level. The thickening of the myocardium is described by the positive strain value of GRS. Treating physicians were blinded for the strain measurements.

### CMR imaging protocol

CMR was performed at 2 different time points: before the start of anthracycline (in early-stage patients) or before the start of trastuzumab treatment (advanced-stage patients), and after 6 m trastuzumab treatment in all (Fig. 1, Supplementary). CMR examinations were performed with a 1.5-T Achieva Intera scanner (Philips Medical Systems; Best; The Netherlands) applying a standard protocol with validated sequences. Ventricular dimensions and function were assessed with an ECG-gated steady-state free-precession cine MR sequence (echo time, 1.5 to 1.9 ms, repetition time, 2.6 to 3.9 ms; in-plane resolution, 1.5 to 2.0 mm; slice thickness, 4 to 5 mm; number of retrospectively reconstructed images per cardiac cycle, 30). Steady-state free-precession cine imaging sequences were acquired in the ventricular short-axis plane, covering the heart from the plane of the atrioventricular valves through the cardiac apex.

### Post-processing CMR software

The artificial intelligence-automated CMR software package (Circle Cardiovascular Imaging: cvi version 5.11) applying deep learning was used as post-processing software. Ventricular end-diastolic (EDV) and end-systolic volume (ESV) were measured using the short-axis stack. LVEF was calculated as the difference between EDV and ESV, relative to the EDV. Endocardial and epicardial contours of the left ventricle that were used for GLS and GRS calculation were automatically tracked using still and motion frames at end-systole and end-diastole (Fig. 1). The contours were then checked by two experienced observers, and manually adjusted when necessary. LV contours in the most basal slices were included if > 50% of ventricle wall was visible. Additionally, late gadolinium enhancement (LGE) was assessed. This technique incorporates the administration of relatively inert extracellular gadolinium contrast during gradient-echo inversion recovery imaging.

### Inter- and intra-observer variability

Inter- and intra- observer variability of 2DE-ST and CMR was not assessed as manually traced borders were checked by two experienced observers. Consensus was reached between the two observers regarding the traced borders that were used for GLS and GRS calculation.

### Cardiotoxicity

Cardiotoxicity was defined as LVEF < 45% during the 6 m follow-up and/or an absolute LVEF decline of > 10% relative to the measurement at study start and measured with CMR – these thresholds are used by the National Cancer Research Institute as definition to interrupt trastuzumab treatment and start ACE inhibitors [23] – and/or any cardiac event for which the patient was hospitalized, including atrial fibrillation, unstable angina pectoris, acute coronary syndrome, and symptomatic heart failure.

### Statistical analyses

Categorical baseline data are presented as numbers and percentages. Shapiro-Wilk tests were used to evaluate the normality of continuous baseline data. Normal distributed data were then expressed as mean values ± standard deviation (SD), and non-normal distributed data as median values with interquartile range (IQR).

Nonlinear mixed effects (NLME) models were used to evaluate changes in 2DE-ST and CMR over time. Pearson’s correlation coefficients for repeated measurements were determined to assess the correlation between 2DE-ST and CMR. Agreement was assessed with the method of Bland-Altman, likewise using (all available) repeated measurements. The limits of agreement were defined as the mean difference ± 1.96 SD.

Linear regression analysis was applied to evaluate the association between 2DE-ST strain at different time points and CMR-based LVEF after 6 m trastuzumab treatment. Multivariable linear regression analyses were then applied to evaluate the added value of 2DE-ST strain to 2DE-LVEF measurements on CMR-based LVEF after 6 m trastuzumab treatment. Results of these regression analyses are expressed as the effect on CMR-LVEF per 1 unit difference in the strain value, with its corresponding 95% confidence interval (CI). We also present the corresponding fraction explained variance (R^2^).

Logistic regression analysis was used to evaluate the association between 2DE-ST strain at different time points and cardiotoxicity. Results are expressed as odds ratios (ORs) with its corresponding 95% CI.

Data analyses were performed using SPSS software, version 24.0 (SPSS, IBM, Chicago, Illinois, USA) and R statistical software (version 3.4.3), in particular the packages “blandr”, “rmcorr” and “lme”. Statistical significance of all tests was set at a two-tailed *p*-value of less than 0.05.

## Results

### Patients characteristics

A total of 83 patients with HER2-positive breast cancer undergoing trastuzumab treatment signed informed consent for their participation in this study. However, 4 patients only received 1 cycle of trastuzumab, while in 25 patients the baseline CMR remained unperformed, and another 7 had poor 2DE-STE image quality. Hence, 47 patients were available for the current analysis. Median age at inclusion was 57 years (IQR 50, 63 years) (Table 1). A total of 38 patients (81%) had early-stage breast cancer and the remaining 9 (19%) had advanced-stage breast cancer.

**Table 1:** Baseline characteristics of the study patients (*n* = 47)

Age, years	57.0 (50.0, 63.0)
	55.0 (10.1)
BMI, kg/m^2^	24.5 (23.1, 29.4)
	25.9 (4.8)
Breast cancer	
Early-stage	38 (81)
Advanced-stage	9 (19)
Anthracycline-based chemotherapy	38 (81)
Left-sided radiotherapy	12 (26)
*Cardiovascular risk factors*	
Hypertension	17 (36)
Diabetes mellitus	3 (6)
Hypercholesterolemia	7 (15)
Positive family history	15 (32)
Current or ever smoker	13 (28)
*Cardiac condition before treatment*	
Valve insuffiency	0 (0)
Arrhytmia	1 (2)
MI/CABG/PCI	0 (0)
*Cardiovascular medication*	
Beta-blockers	1 (2)
ACE inhibitors	2 (4)
Both	1 (2)
*CMR imaging parameters*	
LVEF, %	60.4 (55.8, 66.0)
	60.6 (7.3)
GLS, %	-18.7 (−20.1, −16.9)
	−18.1 (5.6)
GRS, %	30.1 (24.5, 32.9)
	29.5 (5.8)
Left ventricular mass, g	73.6 (65.4, 88.0)
	76.3 (15.2)
Length left ventricle diastolic phase, mm	86.0 (81.5, 89.5)
	85.9 (6.6)
LGE, %	6.0 (5.0–8.0)
	6.9 (3.1)
*2DE parameters*	
LVEF, %	66.0 (63.0, 73.0)
	67.5 (6.5)
ST-LVEF, %	57.2 (53.3, 62.6)
	56.9 (8.1)
ST-GLS, %	−18.8 (− 20.6, −16.3)
	−18.4 (3.0)
ST-GRS, %	21.4 (13.5, 34.1)
	23.6 (13.1)

All continuous variables are shown as median + IQR, mean + SD

Abbreviations: *BMI* body mass index, *MI* myocardial infarction, *CABG* coronary arterial bypass grafting, *PCI* percutaneous coronary intervention, *ACE* angiotensin converting enzyme, *CMR* cardiac magnetic resonance imaging, *2DE* two-dimensional echocardiography, *LVEF* left ventricular ejection fraction, *GLS* global longitudinal strain, *GRS* global radial strain, *LGE* late gadolinium enhancement

### STE measurements

**Fig. 2:**
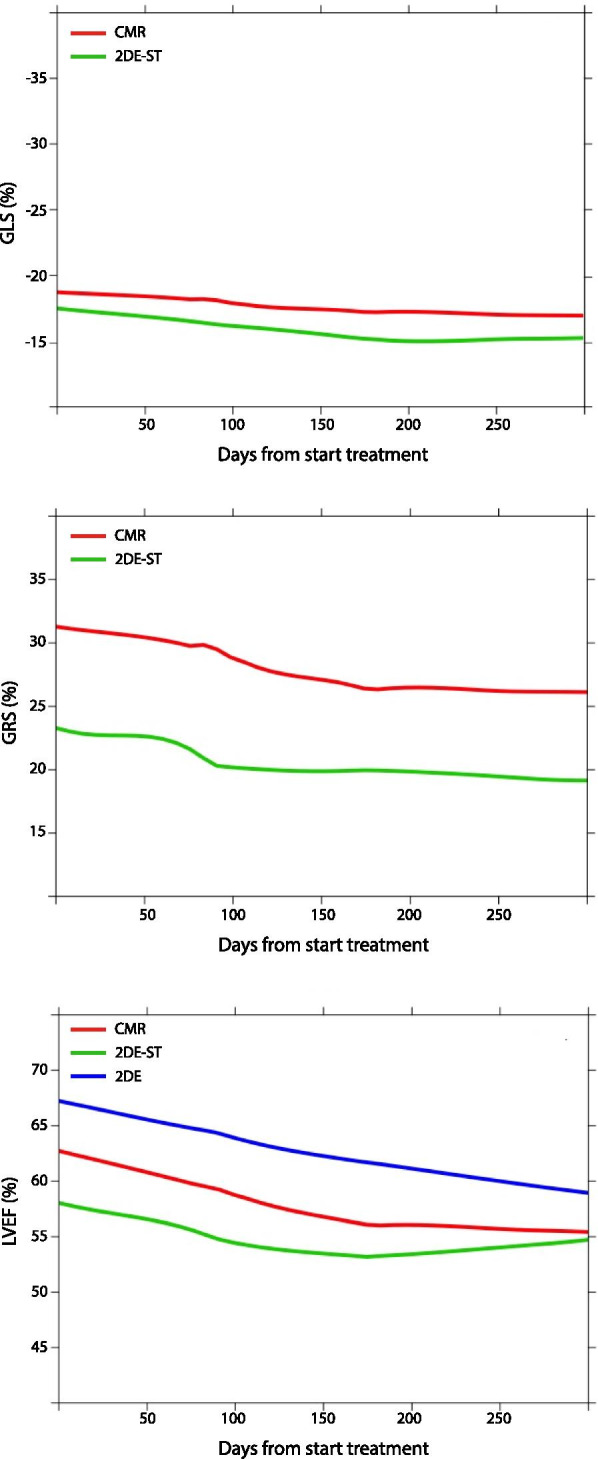
Temporal evolution of 2DE-ST, 2DE and CMR parameters during follow-up. Abbreviations: *2DE-ST* two-dimensional speckle tracking echocardiography, *CMR* cardiac magnetic resonance imaging, *LVEF* left ventricular ejection fraction, *GLS* global longitudinal strain, *GRS* global radial strain, *2DE* two-dimensional echocardiography

2DE-ST was available for all patients at baseline, for 44 patients (94%) after 3 m trastuzumab treatment and for 42 patients (89%) after 6 m trastuzumab treatment (Table 1, Supplementary). At baseline, median LVEF was 57.2% (IQR 53.3, 62.6%), GLS -18.8% (− 20.6, − 16.3%) and GRS 21.4% (13.5, 34.1%), respectively (Table 1). During trastuzumab treatment, the mean LVEF declined with − 0.47%-points per month (95% CI − 0.74%-points, 0.21%-points; *p* < 0.001), whereas the GLS increased with 0.27%-points per month (0.17%-points, 0.38%-points; *p* < 0.001). The mean change in GRS was statistically non-significant (− 0.39%-points per month; 95% CI − 0.80%-points, 0.03%-points; *p* = 0.070). The course of all 2DE-ST parameters during follow-up are shown in Fig. 2.

### CMR measurements

CMR images were available for all patients (*n* = 47) at baseline and for 40 patients (85%) after 6 m trastuzumab treatment. At baseline, median CMR-LVEF was 60.4% (IQR 55.8, 66.0%), GLS − 18.7% (− 20.1, − 16.9%) and GRS 30.1% (24.5, 32.9%)(Table 1). During trastuzumab treatment, the mean LVEF declined with − 0.78%-points per month (95% CI − 1.11%-points, − 0.44%-points; *p* < 0.001), GLS increased with 0.24%-points per month (95% CI 0.14%-points, 0.32%-points; *p* < 0.001) and GRS declined with − 0.68%-points per month (95% CI − 0.94%-points, 0.42%-points; *p* < 0.001). No LGE nor edema was observed during trastuzumab treatment. The course of all CMR parameters during follow-up are shown in Fig. 2.

### Correlations and agreement between 2DE-ST and CMR

**Fig. 3:**
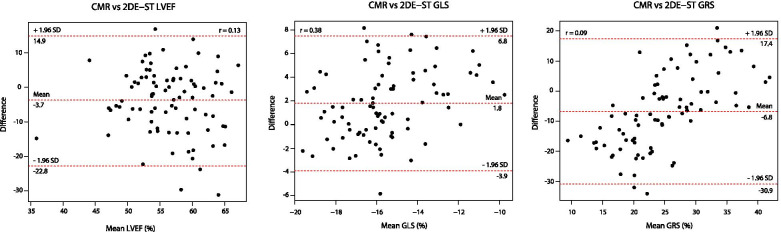
Correlation and agreement between CMR and 2DE-ST. Difference was calculated as 2DE-ST minus CMR. Abbreviations: *CMR* cardiac magnetic resonance imaging, *2DE-ST* two-dimensional speckle tracking echocardiography, *LVEF* left ventricular ejection fraction, *GLS* global longitudinal strain, *GRS* global radial strain

For the analysis of the correlation and agreement between 2DE-ST and CMR, a total of 87 combined baseline and 6 m measurements were available. Agreement with respect to LVEF was poor (Fig. 3). 2DE-ST-GLS and CMR-GLS showed a significant, but weak correlation (*r* = 0.38; *p* < 0.001). The mean difference was 1.8% (2DE-ST-GLS of − 14.7% versus CMR-GLS of − 16.5%), which was statistically significant (*p* < 0.001). However, the limits of agreement were wide, ranging from − 3.9 to 7.5%, suggesting great interindividual variation. We found no significant correlation for GRS based on both methods (*r* = 0.09; *p* = 0.331) and agreement was poor.

### Predictive value of 2DE-ST strain for CMR-based LVEF and cardiotoxicity

Table 2 presents the relations between early 2DE-ST measurements and later CMR-LVEF. 2DE-LVEF before start of trastuzumab and after 3 m trastuzumab treatment were predictive of CMR-LVEF at 6 m trastuzumab treatment. For example, a 1%-point difference in 2DE-LVEF before start of trastuzumab was related with a mean difference of 0.85%-points in CMR-LVEF at 6 m trastuzumab treatment (95% CI 0.42%-points, 1.27%-points; *p* < 0.001). Early 2DE-LVEF values during anthracycline treatment, as well as change values at 3 m trastuzumab treatment, failed to predict CMR-LVEF changes.

**Table 2:** Association between measures obtained by 2DE-ST before anthracycline, before trastuzumab and after 3 months trastuzumab, and CMR-LVEF at 6 months

2DE	CMR-LVEF at 6 months after start trastuzumab						Change in CMR-LVEF at 6 months after start trastuzumab			Cardiotoxicity^**a**^				
	Univariable analysis			Multivariable analysis			Univariable			Univariable				
	Mean difference (95% CI)	***P***-value	R^**2**^	Mean difference (95% CI)	***P***-value	R^**2**^	Mean difference (95% CI)	***P***-value	R^**2**^	Median in patients with CTOX [IQR]	median in patients without CTOX [IQR]	OR (95% CI)	***P***-value	R^**2**^
*Before start anthracycline*														
LVEF, %	0.42 (−0.02, 0.85)	0.058	0.11				0.23 (− 0.22, 0.68)	0.297	0.04	63.0 [58.8, 65.5]	69.0 [64.0, 77.0]	0.77 (0.61, 0.97)	0.027	0.26
ST-GLS, %	0.24 (−0.83, 1.31)	0.649	0.01				0.42 (− 0.64, 1.49)	0.422	0.02	−17.0 [− 18.5, − 14.6]	− 16.0 [− 18.5, − 13.0]	0.81 (0.59, 1.11)	0.377	0.05
ST-GRS, %	0.11 (− 0.11, 0.33)	0.322	0.01				− 0.03 (− 0.26, 0.19)	0.767	0.09	26.5 [15.2, 36.0]	27.2 [13.4, 36.5]	0.99 (0.93, 1.05)	0.721	0.01
*Change during anthracycline*														
LVEF, %	0.11 (− 0.29, 0.51)	0.570	0.01	0.03 (− 0.39, 0.45)	0.879	0.07	−0.05 (− 0.45, 0.35)	0.790	0.01	−2.0 [−7.0, 1.0]	−7.0 [− 13.0, 0.0]	1.07 (0.95, 1.21)	0.250	0.05
ST-GLS, %	− 0.86 (− 2.02, 0.30)	0.142	0.07	− 0.83 (− 2.08, 0.42)	0.186		−0.86 (−2.03, 0.30)	0.141	0.07	4.4 [0.0, 6.0]	0.2 [−0.1, 2.0]	1.39 (0.99, 1.95)	0.058	0.12
ST-GRS, %	−0.04 (− 0.39, 0.31)	0.829	0.00				0.08 (− 0.27, 0.43)	0.631	0.01	−4.2 [− 14.0, 2.3]	−0.9 [− 12.2, 4.3]	1.00 (0.92, 1.10)	0.942	0.00
*Before start trastuzumab*														
LVEF, %	0.85 (0.42, 1.27)	< 0.001	0.30	0.87 (0.41, 1.34)	< 0.001	0.30	0.32 (− 0.16, 0.80)	0.189	0.05	60.0 [57.3, 62.8]	63.0 [60.0, 66.0]	0.88 (0.75, 1.02)	0.080	0.13
ST-GLS, %	− 0.42 (− 1.31, 0.46)	0.337	0.03	0.14 (− 0.68, 0.95)	0.738		−0.28 (− 1.14, 0.58)	0.519	0.01	−13.7 [− 16.9, − 11.2]	− 15.0 [− 17.3, − 12.2]	1.13 (0.87, 1.46)	0.365	0.03
ST-GRS, %	0.08 (− 0.13, 0.28)	0.464	0.07				−0.02 (− 0.22, 0.18)	0.831	0.00	31.4 [4.5, 42.1]	22.4 [8.5, 35.5]	0.99 (0.94, 1.05)	0.830	0.00
*3 Months after start trastuzumab*														
LVEF, %	0.59 (0.30, 0.88)	< 0.001	0.32	0.56 (0.24, 0.87)	0.001	0.35	0.29 (− 0.04, 0.61)	0.080	0.08	55.0 [43.4, 62.8]	60.0 [57.0, 63.0]	0.85 (0.74, 0.98)	0.029	0.28
ST-GLS, %	− 1.14 (− 2.07, − 0.19)	0.018	0.10	− 0.46 (− 1.34, 0.41)	0.288		−0.62 (− 1.54, 0.30)	0.179	0.05	−11.6 [− 15.3, − 9.4]	− 14.1 [− 16.6, − 11.7]	1.36 (0.94, 1.84)	0.073	0.13
ST-GRS, %	0.04 (− 0.23, 0.31)	0.751	0.00				−0.15 (− 0.40, 0.10)	0.237	0.04	28.1 [8.5, 35.3]	21.2 [12.1, 32.0]	1.03 (0.95, 1.11)	0.532	0.02
*Change at 3 months after start trastuzumab*														
LVEF, %	0.30 (−0.11, − 0.71)	0.144	0.06	0.33 (− 0.06, 0.72)	0.094	0.22	0.21 (−0.19, 0.61)	0.292	0.03	−11.0 [− 19.0, − 2.0]	− 4.0 [− 7.0, 0.0]	0.90 (0.80, 1.01)	0.079	0.09
ST-GLS, %	− 1.17 (− 2.14, − 0.20)	0.019	0.14	−1.20 (− 2.16, − 0.24)	0.016		−1.10 (−2.02, − 0.18)	0.021	0.14	5.2 [2.8, 7.0]	1.7 [−0.2, 3.0]	1.81 (1.11, 2.93)	0.016	0.34
ST-GRS, %	− 0.17 (− 0.57, 0.23)	0.386	0.03				−0.12 (− 0.52, 0.27)	0.521	0.02	−0.7 [− 10.0, 2.0]	−3.0 [− 9.7, 1.4]	1.03 (0.92, 1.15)	0.610	0.01

Abbreviations: *2DE* two-dimensional echocardiography, *CMR* cardiac magnetic resonance imaging, *GLS* global longitudinal strain, *GRS* global radial strain, *LVEF* left ventricular ejection fraction, *OR* odds ratio, *R*^2^ explained variance, *ST* speckle tracking, *CTOX* cardiotoxicity

^a^Cardiotoxicity was defined as LVEF < 45% during the 6 months follow-up and/or an absolute LVEF decline of > 10% relative to the measurement at study start and measured with CMR

Patients with higher 2DE-ST-GLS at 3 m trastuzumab treatment demonstrated significantly lower CMR-LVEF at 6 m trastuzumab treatment, but significance was lost after adjustment for 2DE-LVEF. In contrast to 2DE-LVEF, 2DE-ST-GLS change values at 3 m trastuzumab treatment were predictive of CMR-LVEF changes at 6 m trastuzumab treatment. Sensitivity analyses in patients with early-stage breast cancer and advanced-stage breast cancer showed similar results (Table 2, Supplementary).

**Fig. 4:**
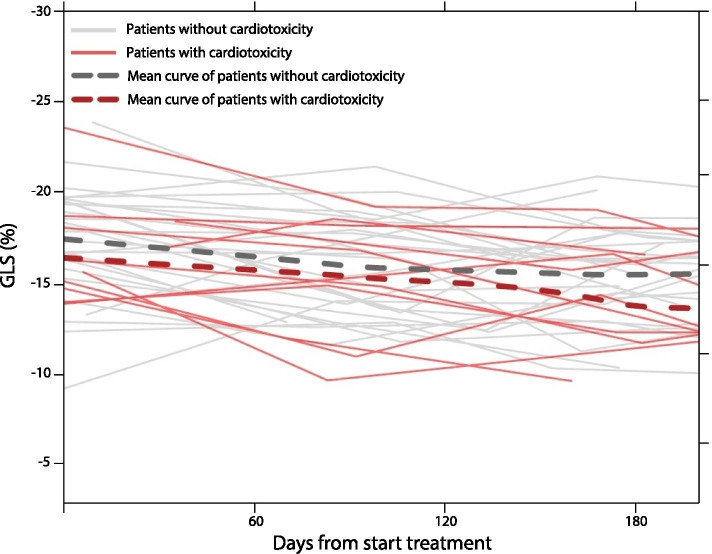
Trajectory of GLS of patients with and without cardiotoxicity. Abbreviations: *GLS* global longitudinal strain

Importantly, 11 patients (28%) developed cardiotoxicity, of whom all experienced an absolute LVEF decline > 10%-points from baseline and 3 patients additionally reached an LVEF below 45%. These patients who developed cardiotoxicity had a median GLS of − 15.2% at baseline, which was not statistically different from the median GLS of − 16.8% at baseline of patients who did not developed cardiotoxicity (*p* = 0.674). In addition, a larger 2DE-GLS change at 3 m trastuzumab treatment was observed in those who developed cardiotoxicity than in those who did not (median 5.2%-points versus 1.7%-points, *p* = 0.036) (Table 2). The odds ratio for a 1%-point difference in change was 1.81 (95% CI 1.11, 2.93; *p* = 0.016). The explained variance of the latter model was 0.34, indicating a moderate effect. Finally, the trajectory of GLS of patients with and without cardiotoxicity showed a trend for a higher GLS increase per month in patients with cardiotoxicity compared to patients without cardiotoxicity (median 0.65%-points versus 0.20%-points, *p* = 0.181) (Fig. 4).

## Discussion

In a broad range of clinical practices, 2DE remains the most obvious imaging modality for the evaluation of therapy-related cardiotoxicity in oncology patients [24]. Nevertheless, 2DE only has a moderate to poor agreement with gold standard CMR regarding the evaluation left ventricular function [4]. We demonstrated that speckle tracking improved the performance of 2DE to predict LVEF changes in HER2-positive breast cancer patients receiving trastuzumab. In particular, cardiotoxic changes could be predicted with greater accuracy, although there is room for further improvement.

Studies on the correlation between 2DE-GLS and CMR-GLS, and 2DE-ST-LVEF and CMR-LVEF showed a wide variation [10–17, 25]. Reported correlation coefficients range from 0.16 in a series of 10 heart transplant recipients to 0.89 in a similar small number of patients with aortic valve stenosis (Table 3, Supplementary). In general, correlation analyses in the field are hampered by small sample sizes, so that estimates are surrounded by uncertainty. That aside, it seems that stronger correlations are reported by studies that included heterogeneous populations of patients undergoing ‘clinically indicated’ echocardiography or CMR, who agreed to undergo the other imaging modality too [14, 15]. Some of these studies even combine observations in patients and healthy volunteers [10, 11, 17]. In general, weaker correlations are reported in studies that focussed on specific, homogeneous populations (including ours). It is well-known that spurious (ly strong) correlations can occur when groups are pooled with differences in absolute values of the variable of interest [26]. For example, the study of Amzulescu et al. reported a high intraclass correlation coefficient (ICC) of 0.89 in a combined series of healthy volunteers (mean 2DE-GLS − 21%), and patients with aortic stenosis (mean 2DE-GLS − 18%), hypertrophic cardiomyopathy (mean 2DE-GLS − 15%), ischemic heart disease (mean 2DE-GLS − 14%) or non-ischemic dilated cardiomyopathy (mean 2DE-GLS − 12%), whereas correlations in the separate subgroups were less convincing [10]. We believe that individual-patient meta-analyses of available datasets are warranted to obtain reliable estimates in relevant target groups. Such analyses are also useful to study reported inter-software variability with respect to strain calculations in more detail [27].

We found only a very weak correlation between 2DE and CMR with respect to GRS. Indeed, in most studies, correlations for GRS were weaker than for GLS (Table 3, Supplementary) [14, 17]. This might be due to the difficulty of epicardial border tracking in 2DE images, and due to the fact that apical views are more suitable for tracking speckles in the longitudinal direction, than in the radial direction [28]. Additionally, a trend to lower 2DE-ST measured LVEF was observed compared to 2DE-LVEF (Fig. 2). This can be explained by differences between the two techniques leading to an underestimation of the LVEF measured with 2DE-ST. Underestimation of the LVEF by 2DE-ST has been previously described when comparing 2DE-ST with 3DE, although a clear explanation is still missing [29]. 

Interestingly, the subgroup analysis in patients without prior anthracycline exposure showed that early GLS change was not associated with CMR-LVEF at 6 months or a change in CMR-LVEF after 6 months trastuzumab (Supplementary Table S2). This could be explained by the fact that non-anthracycline based trastuzumab treatment is associated with much lower cardiotoxicity (cardiotoxicity incidence of 3–7% versus 27%) [30]. As prior anthracycline exposure is an important risk factor for developing trastuzumab-induced cardiotoxicity [31, 32], it might be useful to consider including only patients with prior anthracycline exposure before trastuzumab treatment for future studies.

Our observation that a GLS decline measured with 2DE is related to a subsequent lower CMR-based LVEF (and cardiotoxicity) corresponds with previous studies and meta-analyses [18–21, 33]. Hence, change values appear to contain prognostic information. Accordingly, the American Society of Echocardiography (ASE) and European Association of Cardiovascular Imaging (EACVI) recommend that acquired GLS during chemotherapy should be compared with baseline values [7]. Based on the results of our study, it seems reasonable to add speckle tracking to the 2DE protocol for the regular cardiac surveillance of cancer patients before and during trastuzumab therapy. Importantly, the same modality should be used for serial cardiac surveillance to avoid pitfalls introduced by limited agreement between the modalities [7]. In our follow-up scheme that is based on the current guideline for cardiac monitoring of HER2-positive breast cancer patients during adjuvant or metastatic trastuzumab treatment [34], an abnormal GLS measured with 2DE preceded a LVEF decline by about 3-months. This may provide a window of opportunity to start early cardio-protective therapy. In a small series of HER2-positive breast cancer patients, the SAFE-HEART study recently confirmed that trastuzumab can be safely continued in those with compromised cardiac function, provided that cardiac treatment is timely installed [35]. More recently, the 1 year-results of the prospective multicenter SUCCOUR trial showed that a GLS-guided cardio-protective treatment strategy reduced the incidence of cardiotoxicity, defined as LVEF decline > 10 from baseline to < 55%, compared to a LVEF-guided cardio-protective treatment strategy (5.8% versus 13.7%, *p* = 0.02) [36].

Finally, 3DE-ST may potentially have superior tracking quality over 2DE-ST, as speckles can be tracked in all possible directions and through-plane motion will be absent. It is true that several studies report stronger correlations for GLS and GRS between 3DE-ST and CMR than 2DE-ST and CMR [11, 14]. However, aside from the fact that these studies studied heterogeneous populations, which hampers the interpretation of the findings, it must be realized that the accuracy of 3DE-ST strongly depends on operator experience [26], more so than with 2DE-ST. Unfortunately, in this study we were unable to perform 3DE-ST to study the correlations with CMR in this specific population. Additional studies with larger numbers of participants are required before this technique can be implemented into daily clinical practice.

### Limitations

Several limitations have to be taken into account when interpreting the results of this study. First, we performed a single-center study. Although this center is representative for large, secondary, teaching hospitals, we were unable to study external validity of our findings. Secondly, the sample size was small, although similar to other studies in the field. Consequently, the power was limited to study the additive predictive value of 2DE-derived GLS and LVEF in greater detail. In addition, due to the small numbers of events (*n* = 11), multivariable modelling of predictors for cardiotoxicity was not possible. Lastly, most patients included in our study were diagnosed with early-stage breast cancer for which they were treated with anthracycline and sequential trastuzumab. As these patients only received CMR before anthracycline and not before trastuzumab treatment, we could not investigate the effect of strain on the LVEF change during trastuzumab treatment only. Sensitivity analyses in the early-stage breast cancer patients and advanced-stage breast cancer patients showed, despite the small numbers, similar results.

## Conclusions

In our series of patients with HER2-positive breast cancer with preserved LV function prior to trastuzumab treatment, correlations between 2DE-ST and CMR-derived measurements were weak. Nevertheless, ST appeared to be useful to improve the performance of 2DE to predict detrimental LVEF changes during 6 months trastuzumab treatment, but much remains to be done.

## Supplementary Information

**Additional file 1: Supplementary Figure S1.** Study procedures. **Supplementary Table S1.** All parameters during follow-up. **Supplementary Table S2.** Sensitivity analyses in early-stage breast and advanced-stage breast cancer. **Supplementary Table S3.** Overview of studies investigating the correlation of CMR and 2D-STE in measuring strain.

## Data Availability

The datasets during and/or analysed during the current study available from the corresponding author on reasonable request.

## Abbreviations

2DE: Two-dimensional echocardiography
ST: Speckle tracking
LVEF: Left ventricular ejection fraction
HER2: Human epidermal growth factor receptor 2
2DE-ST: Two-dimensional speckle tracking echocardiography
CMR: Cardiac magnetic resonance imaging
GLS: Global longitudinal strain
GRS: Global radial strain
3DE: Three-dimensional echocardiography
EDV: End-diastolic volume
ESV: End-systolic volume
LGE: Late gadolinium enhancement
ACE inhibitors: Angiotensin converting enzyme inhibitors
SD: Standard deviation
IQR: Interquartile range
NLME: Nonlinear mixed effects
CI: Confidence interval
ICC: Intraclass correlation coefficient
ASE: American Society of Echocardiography
EACVI: European Association of Cardiovascular Imaging
