# Supplementary material for: 2D-echocardiography vs cardiac MRI strain: a prospective cohort study in patients with HER2-positive breast cancer undergoing trastuzumab
Source: Cardiovasc Ultrasound. 2021 Nov 9;19:35. doi: 10.1186/s12947-021-00266-x (PMC8576921; doi:10.1186/s12947-021-00266-x)
Supplement: Supplementary file 1 — Additional file 1: Supplementary Figure S1. Study procedures. Supplementary Table S1. All parameters during follow-up. Supplementary Table S2. Sensitivity analyses in early-stage breast and advanced-stage breast cancer. Supplementary Table S3. Overview of studies investigating the correlation of CMR and 2D-STE in measuring strain. [file 12947_2021_266_MOESM1_ESM.docx]

Supplementary material to Bouwer NI, Liesting C, Kofflard MJM et al. **2D-echocardiography vs cardiac MRI strain using deep learning: a prospective cohort study in patients with HER2-positive breast cancer undergoing trastuzumab.**

Supplementary Figure S1. Study procedures

**CMR  *or* CMR CMR**

**2DE-ST 2DE-ST 2DE-ST 2DE-ST**

**3 months 3 months 3 months**

**Anthracyclines**

**Trastuzumab/ Paclitaxel**

**Trastuzumab**

***Early-stage***

**Trastuzumab/ Paclitaxel**

**Trastuzumab**

***Advanced-stage***

Participants were studied before chemotherapy and before start trastuzumab at standardized intervals every 12 weeks using echocardiography.

**Abbreviations** CMR: cardiac magnetic resonance imaging, 2D-STE: two-dimensional speckle tracking echocardiography.

Supplementary Table S1. All parameters during follow-up

|  | **CMR** | **2DE-ST** | **2DE** |
| --- | --- | --- | --- |
|  | **Median (IQR)** | **Median (IQR)** | **Median (IQR)** |
| *Before anthracycline (n)* | 38 | 38 | 37 |
| LVEF, % | 60.5 (55.0, 66.1) | 59.6 (53.5, 64.7) | 66.0 (63.0, 75.0) |
| GLS, % | -17.0 (-18.5, -14.9) | -19.0 (-20.7, -16.4) |  |
| GRS, % | 30.9 (24.5, 34.4) | 20.8 (14.2, 33.8) |  |
| *Before start trastuzumab (n)* | 9 | 47 | 41 |
| LVEF, % | 59.4 (56.7, 66.5) | 55.9 (51.0, 59.2) | 62.5 (58.3, 66.0) |
| GLS, % | -16.6 (-17.6, -14.9) | -16.7 (-18.3, -13.9) |  |
| GRS, % | 27.2 (23.9, 30.5) | 23.0 (9.1, 34.3) |  |
| *3 months after start trastuzumab (n)* | NA | 44 | 42 |
| LVEF, % |  | 51.8 (48.5, 56.0) | 59.5 (56.0, 63.0) |
| GLS, % |  | -14.8 (-16.7, -11.7) |  |
| GRS, % |  | 16.4 (9.9 to 30.5) |  |
| *6 months after start trastuzumab (n)* | 40 | 42 | 44 |
| LVEF, % | 54.8 (50.7, 60.6) | 54.7 (49.7, 58.2) | 60.0 (55.0, 64.0) |
| GLS, % | -16.1 (-17.3, -14.4) | -14.9 (-16.9, -12.5) |  |
| GRS, % | 26.7 (22.5, 28.9) | 19.0 (12.0, 27.2) |  |
| *Absolute change baseline – 3 months (n)* | NA | 44 | 42 |
| LVEF, % |  | -4.8 (-10.3, 0.4) | -8.0 (-2.0, -14.0) |
| GLS, % |  | 3.5 (1.5 to 4.9) |  |
| GRS, % |  | -3.7 (-9.7, 1.4) |  |
| *Absolute change baseline – 6 months (n)* | 40 | 42 | 44 |
| LVEF, % | -5.4 (-10.0, -0.2) | -2.7 (-11.3, 3.8) | -6.0 (-0.5, -13.0) |
| GLS, % | 1.5 (0.3, 2.1) | 3.3 (1.0, 5.6) |  |
| GRS, % | -4.5 (-7.5, -0.6) | -3.0 (11.7, 3.0) |  |

**Abbreviations** 2DE-ST: two-dimensional speckle tracking echocardiography, CMR: cardiac magnetic resonance imaging, LVEF: left ventricular ejection fraction, GLS: global longitudinal strain, GRS: global radial strain, 2DE: two-dimensional echocardiography, NA: not applicable

Supplementary Table S2. Sensitivity analyses in early-stage breast and advanced-stage breast cancer

| **2DE** |  | **CMR-LVEF at 6 months** | | | | | | |  | **Change in CMR-LVEF at 6 months** | | |  |
| --- | --- | --- | --- | --- | --- | --- | --- | --- | --- | --- | --- | --- | --- |
|  |  | **Univariable analysis** | | |  | **Multivariable analysis** | | |  | **Univariable** | | |  |
|  |  | **mean difference (95% CI)** | **P-value** | **R^2^** |  | **mean difference (95% CI)** | **P-value** | **R^2^** |  | **mean difference (95% CI)** | **P-value** | **R^2^** |  |
| *Before start anthracycline* | | | | | | | | | | | | |  |
| **Early-stage patients (n=38)** | | | | | | | | | | | | |  |
| LVEF, % |  | 0.42 (-0.02, 0.85) | 0.058 | 0.11 |  |  |  |  |  | 0.23 (-0.22, 0.68) | 0.297 | 0.04 |  |
| ST-GLS, % |  | 0.24 (-0.83, 1.31) | 0.649 | 0.01 |  |  |  |  |  | 0.42 (-0.64, 1.49) | 0.422 | 0.02 |  |
| ST-GRS, % |  | 0.11 (-0.11, 0.33) | 0.322 | 0.01 |  |  |  |  |  | -0.03 (-0.26, 0.19) | 0.767 | 0.09 |  |
| *Before start trastuzumab* | | | | | | | | | | | | |  |
| **Early-stage patients (n=38)** | | | | | | | | | | | | |  |
| LVEF, % |  | 0.79 (0.34, 1.25) | 0.001 | 0.29 |  | 0.85 (0.33, 1.37) | 0.002 | 0.30 |  | 0.23 (-0.30, 0.77) | 0.385 | 0.03 |  |
| ST-GLS, % |  | -0.49 (-1.47, 0.48) | 0.311 | 0.03 |  | 0.22 (-0.74, 1.17) | 0.641 |  |  | -0.26 (-1.25, 0.72) | 0.588 | 0.01 |  |
| ST-GRS, % |  | 0.09 (-0.12, 0.31) | 0.386 | 0.03 |  |  |  |  |  | 0.00 (-0.21, 0.22) | 0.979 | 0.00 |  |
| **Advanced-stage patients (n=8)** | | | |  |  |  |  |  |  |  |  |  |  |
| LVEF, % |  | 0.91 (-0.63, 2.46) | 0.199 | 0.26 |  | 0.89 (-0.93, 2.70) | 0.266 | 0.26 |  | 0.42 (-0.88, 1.71) | 0.464 | 0.09 |  |
| ST-GLS, % |  | 0.52 (-2.33, 3.38) | 0.669 | 0.03 |  | 0.22 (-2.71, 3.15) | 0.853 |  |  | 0.55 (-1.59, 2.68) | 0.555 | 0.06 |  |
| ST-GRS, % |  | 0.06 (-0.79, 0.91) | 0.869 | 0.01 |  |  |  |  |  | -0.08 (-0.72, 0.57) | 0.782 | 0.01 |  |
| *3 Months after start trastuzumab* | | | | | | | | | | | | |  |
| **Early-stage patients (n=38)** | | | | | | | | | | | | |  |
| LVEF, % |  | 0.52 (0.17, 0.88) | 0.006 | 0.24 |  | 0.40 (-0.01, 0.81) | 0.057 | 0.31 |  | 0.21 (-0.19, 0.61) | 0.297 | 0.04 |  |
| ST-GLS, % |  | -1.29 (-2.25, -0.33) | 0.010 | 0.21 |  | -0.85 (-1.91, 0.20) | 0.109 |  |  | -0.84 (-1.84, 0.16) | 0.097 | 0.10 |  |
| ST-GRS, % |  | 0.06 (-0.23, 0.36 | 0.664 | 0.01 |  |  |  |  |  | -0.14 (-0.43, 0.14) | 0.314 | 0.04 |  |
| **Advanced-stage patients (n=8)** | | | |  |  |  |  |  |  |  |  |  |  |
| LVEF, % |  | 0.76 (-0.03, 1.55) | 0.058 | 0.48 |  | 0.70 (-0.31, 1.70) | 0.133 | 0.49 |  | 0.19 (-0.62, 1.00) | 0.587 | 0.05 |  |
| ST-GLS, % |  | 1.33 (-1.70, 4.37) | 0.324 | 0.16 |  | 0.40 (2.65, 3.44) | 0.751 |  |  | 1.05 (-1.24, 3.34) | 0.304 | 0.17 |  |
| ST-GRS, % |  | 0.25 (-0.70, 1.20) | 0.545 | 0.06 |  |  |  |  |  | 0.12 (-0.62, 0.85) | 0.716 | 0.02 |  |
| *Change during anthracycline* | | | | | | | | | | | | |  |
| **Early-stage patients (n=38)** | | | | | | | | | | | | |  |
| LVEF, % |  | 0.11 (-0.29, 0.51) | 0.570 | 0.01 |  | 0.03 (-0.39, 0.45) | 0.879 | 0.07 |  | -0.05 (-0.45, 0.35) | 0.790 | 0.00 |  |
| ST-GLS, % |  | -0.86 (-2.02, 0.30) | 0.142 | 0.07 |  | -0.83 (-2.08, 0.43) | 0.186 |  |  | -0.86 (-2.03, 0.30) | 0.141 | 0.07 |  |
| ST-GRS, % |  | -0.04 (-0.39, 0.31) | 0.829 | 0.00 |  |  |  |  |  | 0.08 (-0.27, 0.43) | 0.631 | 0.01 |  |
| *Change at 3 months after start trastuzumab* | | | | | | | | | | | | |  |
| **Early-stage patients (n=38)** | | | | | | | | | | | | |  |
| LVEF, % |  | 0.13 (-0.34, 0.60) | 0.538 | 0.01 |  | 0.13 (-0.29, 0.56) | 0.526 | 0.28 |  | 0.12 (-0.35, 0.59) | 0.608 | 0.01 |  |
| ST-GLS, % |  | --1.55 (-2.53, 0.56) | 0.003 | 0.27 |  | -1.54 (-2.55, -0.52) | 0.005 |  |  | -1.37 (-2.37, -0.38) | 0.009 | 0.22 |  |
| ST-GRS, % |  | -0.18 (-0.57, 0.22) | 0.367 | 0.03 |  |  |  |  |  | -0..13 (-0.51, 0.26) | 0.510 | 0.02 |  |
| **Advanced-stage patients (n=8)** | | | | | | | | | | | | |  |
| LVEF, % |  | 0.69 (-0.54, 1.91) | 0.219 | 0.24 |  | 0.69 (-0.84, 2.22) | 0.300 | 0.24 |  | 0.06 (-1.00, 1.12) | 0.898 | 0.00 |  |
| ST-GLS, % |  | 0.60 (-2.53, 3.74) | 0.655 | 0.04 |  | -0.02 (-3.52, 3.47) | 0.988 |  |  | 0.31 (-2.09, 2.71) | 0.761 | 0.02 |  |
| ST-GRS, % |  | 0.32 (-1.00, 1.64) | 0.573 | 0.06 |  |  |  |  |  | 0.41 (-0.53, 1.35) | 0.327 | 0.02 |  |
| *Change from start anthracycline to 3 months after start trastuzumab* | | | | | | | | | | | | |  |
| **Early-stage patients (n=38)** | | | | | | | | | | | | |  |
| LVEF, % |  | 0.21 (-0.20, 0.63) | 0.303 | 0.04 |  | 0.07 (-0.34, 0.47) | 0.737 | 0.27 |  | 0.03 (-0.39, 0.45) | 0.894 | 0.00 |  |
| ST-GLS, % |  | -1.54 (-2.54, -0.54) | 0.004 | 0.27 |  | -1.49 (-2.56, -0.41) | 0.009 |  |  | -1.37 (-2.38, -0.35) | 0.010 | 0.22 |  |
| ST-GRS, % |  | -0.17 (-0.57, 0.23) | 0.386 | 0.03 |  |  |  |  |  | -0.12 (-0.52, 0.27) | 0.521 | 0.02 |  |

**Abbreviations** CMR, cardiac magnetic resonance imaging; AC, anthracycline; 2DE-ST, two-dimensional speckle tracking echocardiography; GLS, global longitudinal strain; GRS, global radial strain; 2DE, two-dimensional echocardiography; LVEF, left ventricular ejection fraction; CI, confidence interval, R^2^: fraction explained variance

Supplementary Table S3. Overview of studies investigating the correlation of CMR and 2D-STE in measuring strain

| Study | Population | | Sample size | Correlation  GLS GRS GCS | Compared techniques |
| --- | --- | --- | --- | --- | --- |
| Obokata et al. (2015) | Clinically indicated CMR | | 106 | r=0.83 r=0.69 r=0.90  r=0.87 r=0.82 r=0.88 | 2DE-ST vs. CMR-FT  3DE-ST vs. CMR-FT |
| Pryds et al.  (2019) | Healthy subjects  HFrEF  Aortic valve stenosis  Heart transplantation  Perimyocarditis | (n=10)  (n=10) (n=10)  (n=10) | 50 | Overall  r=0.74 r=0.58 r=0.76  Healthy subjects  r=0.32 r=0.43 r=0.17  HFrEF  r=0.16 r=0.12 r=-0.1  Perimyocarditis  r=0.87 r=0.08 r=0.80  Aortic valve stenosis  r=0.89 r=0.86 r=0.90  Heart transplantation  r=0.15 r=0.41 r=0.79 | 2DE-ST vs. CMR-FT |
| Erley et al.  (2019) | Ischemic heart disease  Non-ischemic heart disease  Clinically indicated CMR | (n=15)  (n=33)  (n=2) | 50 | r=0.71 | 2DE-ST vs. CMR-FT |
| Amzulescu et al. (2017) | Healthy volunteers  Ischemic heart disease  Non-ischemic heart disease Hypertrophic cardiomyopathy  Aortic stenosis | (n=31)  (n=39)  (n=36)  (n=11)  (n=19) | 136 | ICC=0.89 ICC=0.80 | 2DE-ST vs. CMR tagging |
| Amzulescu et al. (2018) | Healthy volunteers  LV dysfunction  LV hypertrophy | (n=29)  (n=63)  (n=29) | 119 | ICC=0.65 ICC=0.55  ICC=0.89 ICC=0.83 | 2DE-ST vs. CMR tagging  3DE-ST vs. CMR tagging |
| Onishi et al.  (2015) | Clinically indicated CMR | | 73 | r=-0.87 r=-0.92 | 2DE-ST vs. CMR-FT |
| Kaku et al.  (2014) | Clinically indicated CMR | | 19 | r=0.87 r=0.61 r=0.78 | 3DE-ST vs. CMR |
| Cho et al.  (2006) | Ischemic heart disease | | 30 | r=0.51 r=0.60 r=0.51 | 2DE-ST vs. CMR tagging |
| Bansal et al.  (2008) | Ischemic heart disease | | 30 | r=0.50 r=0.59 r=0.63 | 2DE-ST vs. CMR tagging |

**Abbreviations** GLS: global longitudinal strain, GRS: global radial strain, GCS: global circumferential strain, CMR: cardiac magnetic resonance imaging, CMR-FT: cardiac MRI feature tracking, 2DE-ST: two-dimensional speckle tracking echocardiography, 3DE-ST: three-dimensional speckle tracking echocardiography, HFrEF: heart failure with reduced ejection fraction, r: Pearson’s R, ICC: intraclass correlations, LV: left ventricular
